# The Role of Worry and Emotional Intelligence in Depression in a Non-Clinical and Subclinical Sample

**DOI:** 10.3390/ejihpe16040048

**Published:** 2026-03-28

**Authors:** Maria Rita Sergi, Aristide Saggino, Michela Balsamo, Leonardo Carlucci, Michela Terrei, Marco Tommasi

**Affiliations:** 1 Department of Psychology and Health Sciences, Pegaso University, 80143 Naples, Italy; 2 Department of Psychology, University of Chieti-Pescara, 66100 Chieti, Italy; a.saggino@unich.it (A.S.); mbalsamo@unich.it (M.B.); michela.terrei@phd.unich.it (M.T.); marco.tommasi@unich.it (M.T.); 3 Department of Human Studies, University of Foggia, 71121 Foggia, Italy; leonardo.carlucci@unifg.it

**Keywords:** worry, depression, emotional intelligence, association, affective symptoms, somatic symptoms, Bayesian regression

## Abstract

Background: Recent data show that approximately 3.8% of the global population has a diagnosis of depression. Understanding psychological risk and protective factors is crucial for improving prevention strategies and mental health interventions. Among these, worry and emotional intelligence (EI) have emerged as relevant, yet they are rarely studied together. To date, no studies that analyzed the relationship between emotional intelligence, worry, and depression have been found. Therefore, this study aims to investigate the association among EI, worry, and depression. Methods: This study included 924 participants (*N* = 806 non-clinical and *N* = 118 subclinical sample with elevated depressive symptoms), with a mean age of M = 25.55 years (SD = 11.38). A total of 118 participants (12.8%) met the criteria for clinical depression based on the BDI-II cut-off. All participants completed the Penn State Worry Questionnaire, the Beck Depression Inventory-II, and the Emotional Intelligence Scale. To examine the relationships among all variables examined, zero-order correlation coefficients were calculated. To investigate the predictive power of EI and worry on depression, Bayesian linear regression was conducted. Results: The results showed significant and positive correlations between worry and depression in both samples. EI showed significant and negative correlations with both depression and worry in both the subclinical sample with elevated depressive symptoms and the non-clinical sample. Finally, worry emerged as the strongest contributor to the somatic dimension of depression in both groups. In the subclinical sample with elevated depressive symptoms, age and Evaluation and Expression of Emotion to Self, along with worry, were the best predictors of somatic symptoms. Conclusions: Our data suggest that higher worry levels are associated with higher levels of depressive symptoms, whereas higher EI was negatively associated with depressive symptoms and may play a potential buffering role. Training programs designed to enhance EI could help mitigate the impact of negative events, improve problem-solving skills, and enhance the expression of one’s own emotions.

## 1. Introduction

Depression is characterized by a pervasive low mood, anhedonia (i.e., a lack of pleasure in most daily activities) and other symptoms such as changes in appetite or weight, sleep disturbances, psychomotor retardation or agitation, fatigue or loss of energy, feelings of guilt or worthlessness, and difficulty concentrating or making decisions (Beck et al., 1996). Cognitive dysregulation is a core feature of depression, encompassing cognitive biases and maladaptive cognitive emotion regulation strategies. Cognitive biases include a negative self-referential process, negative attentional bias toward negative stimuli, negative interpretation of ambiguous stimuli and situations, and enhanced recall of negative events (Balsamo, 2010; Balsamo & Saggino, 2007; Balsamo et al., 2015; Beach & Whisman, 2012; Maurer et al., 2018; Ruan-Iu et al., 2023). Maladaptive cognitive emotion regulation strategies refer to the emotional elaboration of a stimulus, aimed at coping by reducing emotional impact. These strategies are characterized by excessive rumination (dwelling on past negative events), limited distraction, and reduced cognitive reappraisal of negative emotion (LeMoult & Gotlib, 2019; Sarkisian et al., 2019).

Recent epidemiological data indicate that the estimated prevalence of depressive symptoms among adults in the Italian general population was 6% between 2016 and 2019. Additionally, depression ranks as the second-costliest pathology after cardiovascular diseases, and it is projected to become the leading cause of loss of years due to ill health by 2030 (Italian National Institute of Health [ISS], 2022). A recent meta-analysis (Cenat et al., 2022) documents an increase in depression rates, particularly from 2020 onwards, among young adults and adults across North America, Europe, Latin America, and Australia. Globally, approximately 3.8% of the population has a diagnosis of depression, with a higher incidence among females (Chahal et al., 2020; World Health Organization [WHO], 2022). The higher occurrence of depressive symptoms in women is attributed to both psychological and biological factors. Biologically, hormonal fluctuations during specific life periods (e.g., premenstrual phase) and low estrogen levels increase vulnerability. Psychologically, women exhibit a higher number of internalizing behaviors (Akhtar-Danesh & Landeen, 2007; Albert, 2015; Piccinelli & Wilkinson, 2000; Sergi et al., 2023). Italian data further reveal a prevalence of depressive symptoms of 6.6% in the 18–34 age group, 5.6% in the 35–49 age group, and 7.3% in the 50–69 age group. Recent Italian epidemiological data show that 8.3% of women reported experiencing of depressive symptoms, compared with 4.5% of men (ISS, 2023; Statista, 2025).

Age emerges as a salient factor in depression prevalence (Islam & Adnan, 2017; Salk et al., 2017). Several studies document associations between age and depression in both male and female populations (Akhtar-Danesh & Landeen, 2007; Islam & Adnan, 2017; Schaakxs et al., 2017; Stordal et al., 2003), with some showing age as a predictor in clinical samples (Akhtar-Danesh & Landeen, 2007; Islam & Adnan, 2017). However, findings are mixed, potentially due to sample differences or measurement variability (Hwu et al., 1996; Knight, 1984; Salk et al., 2017). An interaction effect between age and gender may account for variability, as depressive symptoms appear to increase more sharply with age in women, possibly due to psychosocial stressors and hormonal changes (Albert, 2015; Salk et al., 2017). Understanding such interactions is critical to elucidate depressive disorder development and progression.

Beyond sociodemographic factors, genetic and family influences play a critical role in the onset of depression. Twin studies estimate the heritability of major depressive disorder at approximately 40% (Kendler et al., 2006). Moreover, early-life events such as childhood trauma and abuse have been consistently linked to increased risk of depression later in life (Heim & Nemeroff, 2001; Gilbert et al., 2009). Neurobiological evidence suggests maltreatment during critical developmental periods can alter brain structures implicated in emotion regulation, heightening vulnerability to depressive disorders (Teicher et al., 2016). Including these factors provides a more comprehensive understanding of depression’s multifactorial etiology.

### The Role of Worry and Emotional Intelligence in Depression

The study of psychological risk factors for depression facilitates understanding its course, prevention, and the development of mental health promotion programs (Thapar et al., 2022). Worry is a maladaptive, future-oriented and persistent cognitive–emotional process focused on potential negative outcomes (Gustavson et al., 2018; Rood et al., 2009; Wells, 1995) that has been consistently identified as a risk factor for depression. Numerous longitudinal studies and meta-analyses have demonstrated its predictive role. For instance, Jacobson and Newman (2017) conducted a meta-analysis of 66 longitudinal studies and found that anxiety symptoms—of which worry is a core feature—predict depressive symptoms. Emotional intelligence (EI) is considered a protective factor against affective disorders, including depression. A recent meta-analysis (Sánchez-Álvarez et al., 2015) found a significant and positive correlation between EI and subjective well-being in a sample of 8520 participants, indicating that emotional competencies help manage stress and promote positive emotions. Furthermore, Mestre et al. (2017) found that higher levels of EI are associated with increased resilience, which facilitates better anxiety symptoms. Moreover, Wapaño (2021) showed that EI positively predicted self-efficacy and resilience while negatively predicting anxiety and depression, indicating that emotionally intelligent individuals are more likely to respond positively to their environment.

Worry, traditionally viewed as an anxiety correlate, has since been conceptualized as an independent construct (Davey et al., 1992). Correlational studies reveal consistent positive, significant associations between worry and depression across clinical and non-clinical samples (Borkovec et al., 1983; Calmes & Roberts, 2007; de Jong-Meyer et al., 2009; Dugas et al., 1997; Hong, 2007, 2013; McEvoy & Brans, 2013; Nolen-Hoeksema, 2000; Koerner & Dugas, 2006; among others). Some studies show that worry predicts depression (De Vlieger et al., 2006; Eisma et al., 2017; Hong, 2007), although others have failed to replicate this finding (Dar et al., 2017; Fergus et al., 2013). Experimental research demonstrates higher depression levels in worry conditions compared to controls (Andrews & Borkovec, 1988; Borkovec et al., 1983).

The Beck Cognitive Behavioral Model of depression emphasizes maladaptive cognitive styles such as worry in maintaining depressive symptoms. The Emotional Contrast Avoidance Model (Llera & Newman, 2010, 2014) suggests worry acts as an emotional regulation strategy buffering against sudden negative emotional spikes, maintaining a relatively constant intrapersonal negativity. This aligns with the Tripartite Model (Clark & Watson, 1991), which posits negative effect as a shared dimension of anxiety and depression, with worry as a transdiagnostic cognitive–emotional mechanism involved in both disorders.

In contrast, EI has been consistently associated with lower levels of affective symptoms (Abdellatif et al., 2017). Different conceptualizations of EI have been proposed in the literature (Tommasi et al., 2023). In the Mayer and Salovey (1997) model, EI is defined as a form of intelligence or mental ability—ability EI—namely the capacity to learn, solve problems, adapt to new situations, and pursue life goals. Ability EI is assessed through maximum-performance instruments. In contrast, trait EI can be defined as a series of self-perceptions about the ability to identify emotions (Petrides & Furnham, 2000, 2001, 2003). Consistent with its personality-based nature, trait EI is measured via typical-performance instruments. A third group of models conceptualized EI as a mixed model, integrating both ability-based and trait-based components. In this context, training programs designed to enhance EI have been suggested to be associated with improved emotion regulation to mitigate the impact of negative events, improve problem-solving skills, and enhance the expression of one’s own emotions (Jahangard et al., 2012).

Regarding the association between EI and depression, several studies have demonstrated negative and significant correlations between the two constructs and the predictive power of EI on depression in both adult and adolescent samples, even after controlling for gender and age (Picconi et al., 2019). Abdellatif et al. (2017) found significant and negative correlations among positive expressivity, attending to emotion, and empathy of EI and depression, measured through the BDI-II, in a sample of patients with depression. These findings are consistent with other studies in which EI predicts depression (Abdollahi et al., 2013; Aradilla-Herrero et al., 2014; Extremera & Fernández-Berrocal, 2006; Fernandez-Berrocal et al., 2006; Lloyd et al., 2012; Sergi et al., 2012; Salguero et al., 2012). Ciarrochi et al. (2002) found significant and negative correlations between managing self emotion of EI and depression, measured through the BDI-II, in a sample of undergraduate students. Another study showed that the following factors of EI—emotional recognition and expression, understanding external emotions, direct cognition of emotions, emotional management, and emotional control of EI—predict depression, measured through the BDI-II, in a sample of adults with depression (Downey et al., 2008). These results were confirmed by Davis and Humphrey (2012), who found significant and negative correlations between the total score of EI and depression in a sample of non-clinical adolescents. The total score of EI was a predictor of depression. More recently, Sergi et al. (2021) found significant and negative correlations between EI and depression, measured through the Teate Depression Inventory (TDI; Balsamo & Saggino, 2013; Balsamo et al., 2014) in a sample of non-clinical adults. The results showed that the social skills factor was the best predictor of depression. Moreover, Vucenovic et al. (2023) found significant and negative correlations among expressing and naming emotion and managing emotions of EI and depression in a sample of high-school students, measured through the Depression Anxiety Stress Scale. Finally, several studies (Andrei et al., 2016; Batselé et al., 2019; Gomez-Baya et al., 2017; Karim & Weisz, 2010; Miller et al., 2020; Moeller et al., 2020; Saklofske et al., 2003; Sergi et al., 2012) found significant and negative correlations between depression and global EI, which served as a predictor of depression in non-clinical samples. These results were further supported in clinical samples (Batool & Khalid, 2009; Korkmaz et al., 2020).

While worry and EI operate at different psychological levels (process vs. trait/ability), their joint evaluation offers a balanced model examining vulnerabilities and resilience factors in depression. This dual approach aligns with the literature suggesting that effective mental health screening and prevention should target both risk mechanisms and protective resources. To date, no studies have been found that examined the relationship between EI, worry and depression, limiting current understanding of their interrelation. Clarifying this relationship between worry, EI, and depression enables clinicians to design target interventions—such as training in emotion regulation and cognitive strategies—that improve patients’ ability to maintain positive affect and reduce maladaptive negative thought patterns (Liu & Ren, 2018). The present study addresses this gap by proposing and testing a three-tiered model that integrates demographic, psychological, and behavioral components contributing to depression risk. This model reflects the multilevel nature of vulnerability and protective factors associated with depression.

Therefore, based on the literature reviewed above, the first aim of this work is to analyze the contribution of psychological risk factors (worry and emotional intelligence), age, and gender to depressive symptoms (affective and somatic) on a sample drawn from the relevant general population. In particular, the first aim is divided into four hypotheses:

**H1a.**  *Worry could be positively associated with both affective and somatic factors of depression.*

**H1b.**  *EI could be negatively associated with both affective and somatic factors of depression.*

**H1c.**  *Worry is expected to contribute to the explanation of both the somatic and affective factors of depression in participants above the BDI threshold and the non-clinical sample.*

**H1d.**  *Worry, EI, age, and gender are expected to be associated with somatic and affective depressive symptoms in both the subclinical and the non-clinical sample.*

The second aim of this work is to study the mean difference between a subclinical sample with elevated depressive symptoms and a non-clinical sample among EI, depression, and worry. We hypothesize that the non-clinical sample will have higher means in EI than the subclinical sample with elevated depressive symptoms, while the participants above the BDI threshold will have higher means than the non-clinical sample. Finally, we hypothesize that there will be no age difference between groups and there will be a higher proportion of females in the subclinical sample with elevated depressive symptoms.

## 2. Materials and Methods

*Participants and Procedure:* A total of 924 participants (67.3% females and 32.5% males; missing = 0.2%) were included in the study on a voluntary basis. The inclusion criteria were as follows: (1) participants fluent in Italian living in Italy; (2) age ≥ 18 years. The survey was administered entirely face to face. The participants were drawn from the general reference population. The mean age of the total sample was 25.55 years (minimum = 18 years and maximum = 70 years; SD = 11.38). Eighteen participants (1.9%) did not declare their age. Participants partially overlapped with those included in Sergi et al. (2021) (N = 1725). The present study addresses different research questions, uses different variables, and applies distinct statistical analyses. No analyses, hypotheses, or results from Sergi et al. (2021) are reused or replicated here. Only a subset of participants overlaps, but the constructs, aims, and analytic strategies are entirely distinct.

The sample was divided into a subclinical sample with elevated depressive symptoms and a non-clinical sample based on the Beck Depression Inventory total score (BDI-II; Beck et al., 1996). Scores ≥ 20 indicate moderate depression. Based on this cut-off, 118 participants met the criteria for elevated depressive symptoms. This threshold is widely used in research to identify subclinical groups with clinically relevant symptom levels, without implying a diagnosis of major depressive disorder. A total of 806 participants formed the non-clinical group.

The non-clinical sample included 529 females (65.6%), with a mean of 25.69 years and an SD of 11.58 years, while the subclinical sample with elevated depressive symptoms included 93 females (78.8%), with a mean of 24.63 years and an SD of 9.92 years.

All participants provided written informed consent prior to participation, in accordance with the Declaration of Helsinki (World Medical Association, 2025). Participants’ anonymity and privacy were guaranteed according to Italian and European privacy laws (Italian law n. 196/2003 and EU GDPR 679/2016, respectively).

The sample was recruited in person by trained professionals in the psychometric and clinical fields, following standardized procedures for psychological data collection, including: (a) standardized instructions for questionnaire administration; (b) controlled testing environments to minimize distractions; (c) individual administration to ensure comprehension and privacy; and (d) adherence to ethical and procedural guidelines for psychological assessment (Evers et al., 2017; Hernández et al., 2020).

The study obtained ethical approval from the Department of Medicine and Aging Science, Italy (number 2223/07.09.2021). The study was conducted between October 2021 and October 2023.

### 2.1. Measures

*Emotional Intelligence Scale:* The Emotional Intelligence Scale (EIS; Schutte et al., 1998; Sergi et al., 2021) assesses EI based on the model of Salovey and Mayer (1990). The instrument has been validated in the Italian population by Sergi et al. (2021), who reported good construct validity, adequate internal consistency, and a clear factorial structure consistent with the original model. The self-report instrument consists of 33 items on a 5-point Likert scale from 1 (“Totally disagree”) to 5 (“Totally agree”). The instrument comprises four factors: Evaluation and Expression of Emotion to Self (α = 0.75); Evaluation and Expression of Emotion to Others (α = 0.76); Social Skills (α = 0.73); and Optimism/Mood Regulation (α = 0.64), all calculated in our study. Example items include: “I am aware of the emotions I experience”, “When I am in a good mood, I find it easy to solve problems”. Example items of the first dimension include: “When my mood changes, I see new possibilities” and “I expect good things to happen”. Example items of the second factor include: “By looking at their facial expressions, I recognize the emotions people are experiencing” and “I am aware of the non-verbal messages other people send”. Example items of the third dimension are: “I am aware of my emotions as I experience them” and “I have control over my emotions”. Finally, example items of the last factor include: “Other people find it easy to confide in me” and “Emotions are one of the things that make my life worth living”.

*Beck Depression Inventory-II*: The Beck Depression Inventory-II (BDI-II; Beck et al., 1996; Sica & Ghisi, 2007) is a self-report instrument used to measure the severity of depression in adults. The instrument has been validated in Italy by Sica and Ghisi (2007), showing strong construct validity and high internal consistency in both clinical and non-clinical samples. The BDI-II consists of 21 items based on the diagnostic criteria for major depressive disorder (MMD) and the instrument comprises two factors: affective (α = 0.80) and somatic symptoms (α = 0.82), as observed in the present study. Example items include: “I feel sad”, “I feel discourage about the future”, “I have as much energy as ever”, and “I feel more restless or wound up than usual”.

*Penn State Worry Questionnaire*: Worry is assessed through the Penn State Worry Questionnaire (PSWQ; Meloni & Gana, 2001; Meyer et al., 1990; Morani et al., 1999). The PSWQ has been validated in the Italian population by Morani et al. (1999) and Meloni and Gana (2001), with excellent reliability and a stable one-factor structure. The self-report instrument consists of 16 items on a 5-point Likert scale from 1 (“Not at all typical of me”) to 5 (“Very typical of me”). This instrument has a total score, deriving from the sum of all items (α = 0.89 in our total sample). Example items include: “If I don’t have enough time to do everything, I don’t worry about it” and “My worries overwhelm me”.

### 2.2. Statistical Analyses

*Descriptive statistics*: Descriptive statistics for the collected data are means, standard deviations, skewness and kurtosis. Skewness and kurtosis values between −2 and 2 indicate a normal distribution of the data (Gravetter & Wallnau, 2014).

*Missing data*: No variable showed more than 5% missing data. Therefore, missing values were replaced using the standard substitution code “999” (Barbaranelli & D’Olimpio, 2007).

*Reliability:* Psychological scales’ internal consistency was assessed using Cronbach’s Alpha.

*Bayesian Pearson correlations:* To examine the relationships among EI, depression and worry, the Bayesian Pearson correlation coefficient is calculated. The Bayesian approach provides a quantification of the strength of evidence for the presence or the absence of an association. In particular, the Bayes factor or BF_01_ was interpreted as the degree to which the data were more likely under the null hypothesis than under the alternative, with values greater than 1 indicating increasing evidence for the absence of a correlation and values below 1 indicating growing support for the presence of an effect (Nuzzo, 2017).

*Comparison of correlations from independent samples*: To compare the independent correlations, test statistic z was calculated to test whether correlations differed significantly between the two groups.

*Bayesian linear regression:* We used Bayesian linear regression to estimate the relationships among variables. This method explicitly models the uncertainty associated with parameter estimates and allows the comparison of multiple models without relying on traditional significance thresholds. Bayesian regression begins with a prior probability distribution that reflects initial knowledge or assumptions about the relationships among variables. When new data *D* are observed for a given model M*j*, the prior is updated via Bayes’ theorem to produce a posterior distribution (Bergh et al., 2021). The extent of the update is quantified using the Bayes factor (BFM*j*), which represents the ratio of the posterior odds to the prior odds (Bishop & Tipping, 2003; Dunson et al., 2007; Gelman et al., 2019; Greenland, 2007; Minka, 2000; Vandekerckhove et al., 2018).

For example, a Bayes factor BF_10_ = 5 means the data are five times more likely under Model 1 than under Model 0. Conversely, BF_10_ = 0.5 (i.e., BF_01_ = 2) means the data are two times more likely under Model 0. Interpretation thresholds include:

- 1–3: weak evidence;
- 3–10: moderate evidence;
- 10–30: strong evidence;
- 30–100: very strong evidence;
- >100: extreme evidence for one model over another.

We used non-informative (default) priors to ensure an objective estimation process.

BFMj= pMj D)1− p(Mj|D)p(Mj)1− p(Mj)

If BF_10_ = 5, it means that the data are 5 times more likely under Model *M*_1_ than under *M*_0_. Conversely, if BF_10_ = 0.50, then $\frac{1}{{BF}_{10}}={BF}_{01}$ = 2, which means that the data are 2 times more likely under *M*_0_ than under *M*_1_. Values in the range of $\frac{1}{3}\;<x\;<1$ indicate moderate evidence for *M*_0_; values in the range of $\frac{1}{3}\;<x\;<\;\frac{1}{10}$ indicate strong evidence for *M*_0_; values equal to $\frac{1}{10}$ indicate very strong evidence for *M*_0_; values < $\frac{1}{100}$ indicate extreme evidence for *M*_0_ (Bergh et al., 2021).

Non-informative priors were adopted to minimize subjective influence and allow the data to drive inference, given the exploratory nature of this study and the absence of prior research on the joint relationship between EI, worry, and depression. This choice ensures transparency and comparability with frequentist approaches, while leveraging the advantages of Bayesian estimation such as credible intervals and Bayes factors for model comparison.

In the model, the EIS dimensions and worry were entered as covariates, while affective and somatic symptoms were specified as dependent variables. Given the unbalanced gender distribution in our sample and the well-documented role of demographic factors in depressive symptoms, both age and gender (0 = females and 1 = males) were included as covariates.

*Bayesian Independent Samples T-Test*: To investigate the mean differences between the non-clinical sample and the subclinical sample with elevated depressive symptoms among EIS dimensions, affective and somatic symptoms and worry were estimated using the Bayesian Independent Samples *T*-Test. Cohen’s δ was estimated to calculate the effect size within the Bayesian framework. Finally, to examine demographic differences between the non-clinical and subclinical samples, a Bayesian Independent Samples *T*-Test was conducted to compare age between groups, while a Bayesian Contingency Test was performed to test differences in gender distribution. BF values < 3 indicate anecdotal evidence, values between 3 and 10 indicate moderate evidence, and values > 10 indicate strong evidence for the alternative hypothesis over the null hypothesis.

All statistical analyses are conducted using JASP version 18.1 (JASP Team, 2023). Test statistic z was calculated using the Psychometrica Calculator (Lenhard & Lenhard, 2014).

## 3. Results

Table 1 shows *occupational categories* in the non-clinical sample and in the subclinical sample with elevated depressive symptoms.

Table 2 presents *descriptive* statistics of EIS dimensions, depression, and worry in the non-clinical sample and subclinical sample with elevated depressive symptoms. All scores showed normal values of skewness and kurtosis. For the subclinical sample with elevated depressive symptoms (*N* = 118), the assumption of univariate normality was assessed by computing standardized skewness and kurtosis values, following the criteria outlined by Kim (2013). Specifically, z-scores were obtained by dividing the skewness and kurtosis values by their respective standard errors. All Z-skewness values were within the acceptable range (absolute z < 3.29) for medium-sized samples, except for the Optimism/Mood Regulation variable, which showed a slight deviation from normality (Z-skewness = 4.49). All Z-kurtosis values were below the critical threshold of an absolute z-value of 3.29, indicating no substantial departure from normality.

In the present study, internal consistency was estimated separately for the non-clinical and subclinical groups. For the non-clinical sample, the EIS showed a Cronbach’s Alpha of 0.84, while in the subclinical sample with elevated depressive symptoms, the Alpha coefficient was 0.85.

Regarding the PSWQ, internal consistency in the non-clinical group was 0.88, compared to 0.87 in the subclinical sample.

Similarly, the BDI-II demonstrated good reliability, with a Cronbach’s Alpha of 0.77 in the non-clinical sample and 0.50 in the subclinical sample with elevated depressive symptoms.

Table 3 presented the associations among EIS dimensions, depression, and worry in the non-clinical sample and the subclinical sample with elevated depressive symptoms. In both groups, the results generally showed significant correlations among the variable investigated. Specifically, in the non-clinical sample, significant and negative correlations were observed between the somatic symptoms of depression and Social Skills (BF_01_ < 0.01), while significant and negative correlations were also found between the affective symptoms of depression and Expression and Evaluation of Emotion to Self (BF_01_ < 0.01).

Worry showed significant and negative correlations with Social skills (BF_01_ < 0.01). Finally, significant and positive correlations were found between worry and affective and somatic symptoms of depression (BF_01_ < 0.01).

In the subclinical sample with elevated depressive symptoms, EI dimensions remained positively interrelated, with several correlations supported by strong evidence (BF_01_ < 0.01 or BF_01_ < 0.03). However, associations between EI and depressive symptoms were attenuated. Affective and somatic symptoms showed no significant correlations with any EI dimension, with Bayes factors indicating substantial evidence for the null hypothesis. Similarly, correlations between EI and worry were small and unsupported by strong evidence. In the non-clinical group, the correlations between worry and depressive symptoms (both affective and somatic) were stronger than in the subclinical group, whereas the negative correlations between the EI dimensions and depressive symptoms were generally weaker.

Table 4 shows the comparison of correlations from independent samples. Overall, most correlations did not differ significantly between the two groups, indicating a comparable pattern of associations among EI, worry, and depressive symptoms.

Table 5 presented Bayesian linear regression for affective symptoms in the non-clinical sample. In this model, the independent variables included Evaluation and Expression of Emotion to Self, Evaluation and Expression of Emotion to Others, Social Skills, Optimism/Mood Regulation, worry, gender, and age, while affective symptoms were the dependent variable. The Bayes factor BF_01_ compares all models to the null model. The best model was worry + Evaluation and Expression of Emotion to Self. These results were confirmed by Table S1, where posterior inclusion probabilities were near 1. Therefore, worry and Evaluation and Expression of Emotion to Self were linked to affective symptoms in the non-clinical sample.

Table 6 presents Bayesian linear regression for somatic symptoms in the non-clinical sample. In this model, the independent variables were Evaluation and Expression of Emotion to Self, Evaluation and Expression of Emotion to Others, Social Skills, Optimism/Mood Regulation, worry, gender, and age, while somatic symptoms were the dependent variable. The Bayes factor BF_01_ compares all models to the null model. Worry and Social Skills were related to somatic symptoms. This result was supported by Table S2, in which posterior inclusion probabilities were near 1. Therefore, worry and Social Skills were associated with somatic symptoms.

Table 7 presents Bayesian linear regression for affective symptoms in the subclinical sample with elevated depressive symptoms. In this model, the independent variables included Expression and Evaluation of Emotion to Self, Evaluation and Expression of Emotion to Others, Social Skills, Optimism/Mood Regulation, worry, gender, and age, while affective symptoms were the dependent variable. The Bayes factor BF_01_ compares all models to the null model. In this model, worry was linked to affective symptoms. These results were confirmed by Table S3, in which posterior inclusion probabilities are near 1.

Table 8 presents Bayesian linear regression for somatic symptoms in the subclinical sample with elevated depressive symptoms. In this model, the independent variables were Expression and Evaluation of Emotion to Self, Evaluation and Expression of Emotion to Others, Social Skills, Optimism/Mood Regulation, worry, gender, and age, while somatic symptoms were the dependent variable. The Bayes factor BF_01_ compares all models to the null model. The best model was gender + age + Expression and Evaluation of Emotion to Self + Expression and Evaluation of Emotion to Others + Social Skills + Optimism/Mood Regulation + worry. These results were confirmed by Table S4, in which posterior inclusion probabilities were near 1. Therefore, age, gender, Expression and Evaluation of Emotion to Self, Expression and Evaluation of Emotion to Others, Social Skills, Optimism/Mood Regulation, and worry were included in the model.

Table 9 demonstrates that all measures, except for Evaluation and Expression of Emotion to Others, showed moderate evidence for difference between groups. Table 1 indicates that the non-clinical sample had higher means in Evaluation and Expression of Emotion to Self, Social Skills, and Optimism/Mood Regulation, while the clinical sample had higher means in affective symptoms, somatic symptoms, and worry. Cohen’s δ = 1.052 indicated a very large effect size. The Bayesian Independent Samples *T*-Test showed moderate evidence for the absence of an age difference between groups (BF_01_ = 5.945), while the Bayesian Contingency Test indicated strong evidence for a difference in gender distribution (BF_01_ = 0.051). Specifically, the subclinical sample with elevated symptoms included 93 females and 23 males, confirming the imbalance between groups.

## 4. Discussions

**Figure 1:**
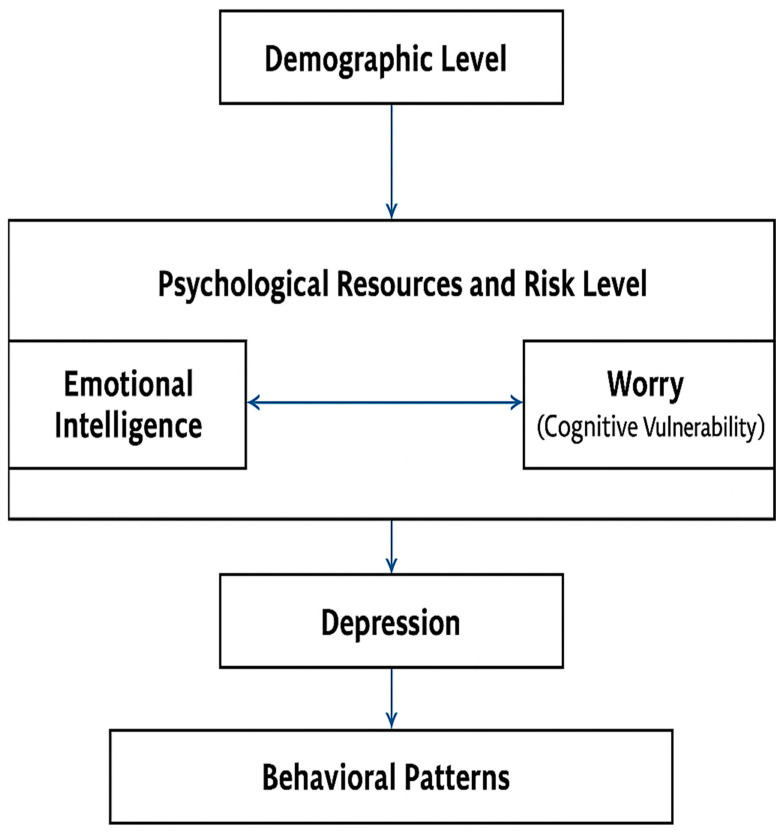
Proposed Multilevel Model of Depression Risk.

The Multilevel Model of Depression Risk conceptualizes depression vulnerability and resilience as involving interrelated demographic, psychological, and behavioral factors (Figure 1). At Level 1 (Demographic Factors), age was related to somatic symptoms in the subclinical group. At Level 2 (Psychological Resources and Risks), EI—particularly the ability to evaluate and express emotion to oneself—was negatively associated with depressive symptoms, suggesting a potential buffering role. Worry was positively associated with depressive symptoms, consistent with its conceptualization as a cognitive–emotional vulnerability correlate (Gustavson et al., 2018; Hong, 2007; Nolen-Hoeksema, 2000; Ruan-Iu et al., 2023). At Level 3 (Behavioral Patterns), the clinical/subclinical sample exhibited increased severity of affective and somatic symptoms concomitant with maladaptive behaviors exemplified by worry and emotional dysregulation. This integration highlights how demographic factors create the base of vulnerability, while EI and worry modulate the risk and manifestation of depressive symptoms.

The existing literature underscores the importance of psychological processes underlying worry and EI in depression’s development and the maintenance of worry (Abdellatif et al., 2017; Clarke et al., 2017; Goodwin et al., 2017; Kotsou et al., 2019; Newman & Llera, 2011). Our findings contribute to the increasing literature emphasizing the significance of investigating the psychological processes in clinical and non-clinical samples that are at risk of developing depressive symptoms (Clarke et al., 2017; Goodwin et al., 2017; Newman & Llera, 2011). This study complements prior work by examining these factors concurrently in Italian adults, marking the first such investigation regionally.

The first aim of this work was to analyze the contribution of psychological risk factors, age, and gender to depression and to examine the associations among worry, EI, and depressive symptoms. Our results showed that worry was positively associated with both affective and somatic symptoms in the non-clinical sample, and the direction of the associations was consistent with the subclinical group, although correlations did not reach statistical significance in this latter sample. These findings align with prior research (Borkovec et al., 1983; de Jong-Meyer et al., 2009; De Vlieger et al., 2006; de Vito et al., 2019; Dugas et al., 1997; Eisma et al., 2017; Goring & Papageorgiou, 2008; Hong, 2007, 2013; Hughes et al., 2008; Manfredi et al., 2011; McEvoy & Brans, 2013; Mourady et al., 2017; Naragon-Gainey et al., 2017; Nolen-Hoeksema, 2000; Perini et al., 2006; Robichaud & Dugas, 2005; Segerstrom et al., 2000; Short & Mazmanian, 2013; Siegle et al., 2004; Sorg et al., 2012; Vickers & Vogeltanz-Holm, 2003; E. R. Watkins & Moulds, 2009).

Moreover, our results demonstrate that EI has, in general, significant and negative correlations with depression and worry, corroborating previous research (Andrei et al., 2016; Aradilla-Herrero et al., 2014; Batool & Khalid, 2009; Ciarrochi et al., 2002; Davis & Humphrey, 2012; Downey et al., 2008; Extremera & Fernández-Berrocal, 2006; Fernandez-Berrocal et al., 2006; Gomez-Baya et al., 2017; Karim & Weisz, 2010; Korkmaz et al., 2020; Lloyd et al., 2012; Moeller et al., 2020; Picconi et al., 2019; Saklofske et al., 2003; Salguero et al., 2012; Sergi et al., 2012; Sergi et al., 2021; Vucenovic et al., 2023; Zysberg & Zisberg, 2022). In the non-clinical sample, higher scores of Evaluation and Expression of Emotion to Self are associated with lower scores of affective and somatic symptoms of depression. Moreover, higher levels of worry are associated with lower Social Skills scores. In the subclinical sample with elevated depressive symptoms, higher scores of Evaluations and Expression of Emotion to Self are associated with lower scores of somatic symptoms of depression, while higher levels of worry are associated with higher scores of Evaluation and Expression of Emotion to Others, even if these correlations did not reach statistical significance. This latter result can be explained by a high fear of other people’s judgement, increasing worry levels.

Furthermore, hypotheses H1c (worry is expected to contribute to the explanation of both the somatic and affective factors of depression in participants above the BDI threshold and in the non-clinical sample) and H1d (worry, EI, age, and gender are expected to be associated with somatic and affective depressive symptoms in both the subclinical and the non-clinical sample) were partially confirmed. Worry contributed to the affective and somatic factor of depression in both samples, while in the subclinical group both psychological dimensions (EI and worry) and demographic factors (age and gender) emerged as the strongest contributors of somatic symptoms of depression. These findings align with prior research (Abdollahi et al., 2013; Andrei et al., 2016; Aradilla-Herrero et al., 2014; Batool & Khalid, 2009; Batselé et al., 2019; Davis & Humphrey, 2012; Extremera & Fernández-Berrocal, 2006; Fernandez-Berrocal et al., 2006; Gomez-Baya et al., 2017; Karim & Weisz, 2010; Korkmaz et al., 2020; Lloyd et al., 2012; Miller et al., 2020; Moeller et al., 2020; Saklofske et al., 2003; Sergi et al., 2012; Salguero et al., 2012).

Finally, our results indicate that the non-clinical sample demonstrated greater abilities in expressing and processing their own emotions, as well as higher levels of optimism and social skills, compared to the subclinical sample. In contrast, the subclinical sample with elevated depressive symptoms exhibited higher levels of somatic and affective symptoms of depression, as well as elevated levels of worry compared to the non-clinical sample.

Our study analyzed the role of risk factors of depression that permit us to develop protective strategies for depression (Abdellatif et al., 2017; Abdollahi et al., 2013; Ciarrochi et al., 2002; Downey et al., 2008; Lloyd et al., 2012; Vucenovic et al., 2023). Higher EI was associated with better understanding and processing of emotions, which in turn was linked to fewer repetitive negative thoughts (E. Watkins & Baracaia, 2001). Indeed, EI was associated with more positive mood states, which have been linked to lower levels of psychopathological symptoms, such as depression (Armstrong et al., 2011; Bauld & Brown, 2009; Di Fabio, 2011; Fernández-Berrocal et al., 2005; Martins et al., 2010; Nielsen-Prohl et al., 2013; Picconi et al., 2019; Schutte & Malouff, 2011; Sergi et al., 2021; Sharif et al., 2013; Slaski & Cartwright, 2003; Yalcin et al., 2008). In particular, the ability to understand one’s emotional states was associated with better adaptation to stressful situations, reducing the impact of negative emotions, and increasing well-being (Abdellatif et al., 2017; Abdollahi et al., 2013; Delhom et al., 2018; Lloyd et al., 2012; Sergi et al., 2021). This scientific evidence could elucidate why the subclinical sample exhibits lower scores in EI factors compared to the non-clinical sample.

Another risk factor for depression was the negative attribution style to daily events and worry (Girgus & Yang, 2015). Our results show that the fixation on negative and uncontrollable future thoughts and images is associated with depression. In particular, the ability to understand and to express one’s own emotions and worry are the best contributors of affective symptoms of depression in the non-clinical sample; when Evaluation and Expression of Emotion to Self and worry are associated with age, they become the best predictor of somatic symptoms of depression. These associations may help contextualize the lower EI scores observed in the subclinical sample. Taken together, our findings highlight the importance of measuring both maladaptive processes, such as worry, and protective factors, such as EI, to advance a more comprehensive understanding and prevention of depression.

Our findings highlight that EI and worry relate to the cognitive and emotional processes of depressive symptoms. Worry was positively associated with depressive symptoms, supporting theoretical models that conceptualize it as a maladaptive cognitive–emotional strategy. Maintaining a chronic state of negative activation may increase physiological tension and reduce attentional flexibility, contributing to the somatic component of depression. Moreover, worry overlaps with repetitive negative thinking, a transdiagnostic mechanism that prolongs negative affect.

Individuals with higher EI are better able to detect early signs of distress and implement cognitive reappraisal strategies. EI reduces reliance on maladaptive strategies such as worry. This supports the conclusion that EI may play a buffering role, mitigating the impact of negative emotional states.

In the subclinical group, the associations were weaker. When depressive symptoms become more intense, cognitive and emotional processes tend to become more rigid, reducing the protective role of EI. Chronic worry and emotional dysregulation may gradually weaken emotional skills, contributing to the lower EI scores observed in individuals with higher depressive symptoms.

Age also contributed to somatic symptoms in the subclinical group, indicating that demographic factors interact with psychological mechanisms. In the multilevel model proposed, demographic characteristics form a background of vulnerability, while worry and EI influence the risk of depressive symptoms.

Our study has several limitations. Firstly, as the Bayesian regression model only examines associations among the variables analyzed in the present research, causality cannot be estimated. Secondly, the limited generalizability of our results to clinical samples is a limitation. The study does not assess the presence of diagnoses or mental-health-related conditions, history of mental disorders/psychological difficulties, or comorbidity with anxiety. Furthermore, there is an imbalance between groups, which may have affected the comparability and generalizability of the results. Although some correlations reached statistical significance at the 95% confidence level, their strength was generally weak. Given the large size, it is possible the statistical significance was driven by the number of observations. Finally, the use of a single-instrument cut-off score (BDI-II) to distinguish between participants with higher and lower depressive symptoms represents a limitation. Dichotomizing a continuous variable such as depression can lead to a loss of statistical power and reduced precision of the results. Future research should consider maintaining depression scores as continuous variables and exploring non-linear relationships with predictors.

Future research should replicate our study with more, different clinical samples and should investigate other potential psychological processes underlying worry and EI in predicting depression. Additionally, future studies could use predictive models of depression with machine learning. Finally, future research will be directed toward experimental studies with emotion regulation training programs, as well as the exploration of mediating and moderating variables such as gender or trauma.

### Practical Implications

The findings hold important implications for prevention and interventions in psychotherapeutic and clinical fields. The interplay between worry and depression might be exacerbated by poor EI characterized by difficulties in expressing, using, and understanding one’s own and others’ emotions. Intervention programs focused on enhancing EI in individuals exhibiting depressive symptoms and excessive worry could bolster their ability to manage these negative emotional experiences. Psychological interventions that focus on identifying and controlling negative emotions, such as anger or sadness, and understanding the relationship between emotions and physical reactions could be particularly effective in promoting EI (Gigantesco et al., 2015; Karahan & Yalçın, 2009). Additionally, the development and use of adaptive coping strategies—like cognitive flexibility, problem-focused coping, and emotion-focused coping—have been linked to lower levels of depressive symptoms (Vucenovic et al., 2023). Indeed, mental health promotion initiatives might integrate emotion management skills training for individuals without formal mental disorders to enhance resilience and emotional regulation, thereby preventing psychological distress. The consistent role of worry highlights the importance of incorporating interventions that directly target repetitive negative thinking. Cognitive–behavioral strategies such as cognitive restructuring and training in attentional flexibility may be especially beneficial for individuals showing early somatic manifestations of distress, even in the absence of clinically significant depressive symptoms (Gigantesco et al., 2015; Evers et al., 2017; McEvoy et al., 2013). Moreover, the negative associations between EI and depressive symptoms, especially the ability to evaluate and express one’s own emotions, suggest that enhancing emotional clarity and expressiveness may serve as a protective factor. Interventions focused on improving emotional awareness, labeling, and communication, such as emotion-focused psychoeducation, mindfulness-based programs, or EI-based training modules, may help individuals regulate negative affect more effectively and reduce reliance on maladaptive strategies such as worry (Gu et al., 2015; Querstret et al., 2020). Furthermore, the finding that EI and age jointly contributed to somatic symptoms in the subclinical group indicates that interventions may need to be tailored according to individual characteristics. Younger adults with elevated depressive symptoms may particularly benefit from structured programs that strengthen emotional competencies and reduce cognitive rigidity associated with chronic worry. Finally, the differences between the non-clinical and subclinical groups suggest that screening for both worry and EI may help identify individuals at risk before depressive symptoms intensify. Integrating brief assessments of repetitive negative thinking and emotional competencies into university counseling services, primary care settings, or community mental health programs could support early detection and timely intervention (Marchetti et al., 2016; Topper et al., 2017).

Additionally, the European Year of Youth has organized new activities for young people and designed new platforms for peer-to-peer relationships among young people (The Lancet Regional Health–Europe, 2022). A final practical implication of our results for practitioners concerns school-based programs that could also focus on risk factors for depression or other psychopathologies, such as bullying (Thapar et al., 2022).

## 5. Conclusions

Our findings are consistent with the Multilevel Model of Depression Risk proposed in the present study, in which higher levels of worry are associated with greater affective and somatic symptoms, supporting its role as a cognitive–emotional vulnerability factor. Conversely, higher ability to evaluate and express one’s own emotions was negatively associated with depressive symptoms, suggesting a potential buffering function.

Worry emerged as the strongest contributor to the somatic dimension of depression in both samples, while in the subclinical group, age and EI also contributed to somatic symptoms (McEvoy & Brans, 2013). The non-clinical sample showed higher emotional competence, optimism, and social skills, whereas individuals with elevated depressive symptoms reported greater worry and more severe affective and somatic symptoms.

Overall, the findings highlight the importance of assessing both maladaptive processes (such as worry) and protective resources (such as EI) to better understand how depressive symptoms develop and persist (Picconi et al., 2019; Sergi et al., 2021; Wapaño, 2021).

In conclusion, our data suggest that higher levels of worry are associated with higher levels of depression. Furthermore, difficulties in understanding, expressing, and regulating emotions correlate with higher levels of worry and depression. In particular, the ability to appraise and express emotions was negatively associated with depressive symptoms and may play a potential buffering role. Therefore, individuals with greater emotional competence are more likely to exert better control over worry, resulting in lower levels of depression.

## Figures and Tables

**Table 1:** Occupational category in the non-clinical sample and in the subclinical sample with elevated depressive symptoms.

Occupational Category	Non-Clinical Sample (%)	Subclinical Sample with Elevated Depressive Symptoms (%)
**Undergraduate students**	75.4	9.92
**Employment**	4.7	n.a.
**Housewives**	3.3	5.9
**Office clerks**	3.2	
**Freelancers**	3.1	2.5
**Workers**	2.9	n.a.
**Teachers**	1.9	n.a.
**Traders**	1.1	n.a.
**Armed forces personnel**	1.0	n.a.
**Technical workers**	0.9	n.a.
**Nurses**	0.6	n.a.
**Doctors**	0.6	0.8
**Social operators**	0.1	0.8
**Other employments**	n.a.	5.9
**Technical employments**	n.a.	2.5
**Missing data**	1.1%	3.4

Note: n.a. = not available.

**Table 2:** Mean, standard deviation, and normality indices of EIS dimensions, depression, and worry in the non-clinical sample and the subclinical sample with elevated depressive symptoms.

Non-Clinical Sample (*N* = 806)	Min	Max	Mean	SD	Skewness	Kurtosis
Evaluation and Expression of Emotion to Self	16	45	35.40	4.54	−0.51	0.51
Evaluation and Expression of Emotion to Others	9	30	22.24	3.78	−0.40	0.10
Social Skills	6	20	14.70	2.90	−0.27	−0.25
Optimism/Mood Regulation	11	25	21.20	2.60	−0.76	0.59
Affective symptoms	0	14	3.31	2.91	0.88	0.28
Somatic symptoms	0	17	4.53	3.19	0.53	−0.06
Worry	16.00	80.00	47.74	10.58	−0.05	−0.08
**Subclinical sample with elevated depressive symptoms (*N* = 118)**						
Evaluation and Expression of Emotion to Self	16	44	33.16	5.43	−0.45	0.32
Evaluation and Expression of Emotion to Others	10	30	21.89	4.87	−0.57	−0.31
Social Skills	5	20	12.72	3.20	0.14	−0.18
Optimism/Mood Regulation	8	25	20.01	3.29	−1.00	1.42
Affective symptoms	5	26	13.12	4.17	0.70	0.60
Somatic symptoms	3	21	13.32	3.39	−0.02	−0.10
Worry	29.00	79.00	59.33	10.66	−0.37	−0.25

Note. Min = Minimum, Max = Maximum, SD = Standard Deviation.

**Table 3:** Bayesian Pearson correlations analysis among EIS dimensions, depression, and worry in both samples.

Non-Clinical Sample (*N* = 806)	1.	2.	3.	4.	5.	6.	7.
1. Evaluation and Expression of Emotion to Self							
2. Evaluation and Expression of Emotion to Others	0.307 ***						
3. Social Skills	0.325 ***	0.338 ***					
4. Optimism/Mood Regulation	0.506 ***	0.373 ***	0.269 ***				
5. Affective symptoms	−0.184 ***	−0.015	−0.127 *	−0.034			
6. Somatic symptoms	−0.081	−0.065	−0.154 ***	−0.002	0.456 ***		
7. Worry	−0.048	−0.042	−0.139 ***	0.110	0.363 ***	0.288 ***	
**Subclinical sample with elevated depressive symptoms** ** (*N* = 118)**	**1.**	**2.**	**3.**	**4.**	**5.**	**6.**	**7.**
1. Expression and Evaluation of Emotion to Self							
2. Evaluation and Expression of Emotion to Others	0.369 ***						
3. Social Skills	0.447 ***	0.365 ***					
4. Optimism/Mood Regulation	0.513 ***	0.518 ***	0.314 **				
5. Affective symptoms	−0.117	0.151	0.001	−0.016			
6. Somatic symptoms	−0.202	−0.011	−0.150	−0.172	0.030		
7. Worry	−0.074	0.211	−0.088	0.083	0.189	0.201	

Note: * BF_01_ < 0.1, ** BF_01_ < 0.03, *** BF_01_ < 0.01.

**Table 4:** Comparison of correlations from independent samples.

	1.	2.	3.	4.	5.	6.	7.
1. Evaluation and Expression of Emotion to Self							
2. Evaluation and Expression of Emotion to Others	−0.702						
3. Social Skills	−1.441	0.309					
4. Optimism/Mood Regulation	−0.095	2.333 **	−0.493				
5. Affective symptoms	−0.688	−0.662	−1.291	−0.181			
6. Somatic symptoms	1.24	−0.543	−0.041	1.722 *	4.636 ***		
7. Worry	0.262	−2.57 *	−0.518	0.273	1.896 *	0.929	

Note: * *p* < 0.5, ** *p* < 0.01, *** *p* < 0.001.

**Table 5:** The best models from the Bayesian linear regression for affective symptoms in the non-clinical sample.

	p(M)	p(M|*D*)	BFM*j*	BF_01_	R^2^
Worry + Expression and Evaluation of Emotion to Self	0.12	0.22	49.32	1.00 × 10^−27^	0.15
Gender + Worry + Expression and Evaluation of Emotion to Self	0.00	0.20	71.16	6.77 × 10^−28^	0.16
Gender + Age + Worry + Expression and Evaluation of Emotion to Self	0.00	0.06	18.19	2.24 × 10^−27^	0.16
Gender + Worry + Expression and Evaluation of Emotion to Self + Evaluation and Expression of Emotion to Others	0.00	0.06	17.81	2.29 × 10^−27^	0.16
Worry + Expression and Evaluation of Emotion to Self + Evaluation and Expression of Emotion to Others	0.00	0.05	16.33	2.48 × 10^−27^	0.16
Age + Worry + Expression and Evaluation of Emotion to Self	0.00	0.05	14.54	2.77 × 10^−27^	0.16
Gender + Age + Worry + Expression and Evaluation of Emotion to Self + Evaluation and Expression of Emotion to Others	0.00	0.03	5.11	7.72 × 10^−27^	0.16
Gender + Worry + Expression and Evaluation of Emotion to Self + Social Skills	0.00	0.03	8.51	4.64 × 10^−27^	0.16
Gender + Worry + Expression and Evaluation of Emotion to Self + Optimism/Mood Regulation	0.00	0.02	6.85	5.74 × 10^−27^	0.16

Note: p(M) = prior model probabilities; p(M|*D*) = posterior model probabilities; BFM*j* = the change from prior to posterior model odds; BF_01_ = Bayes factor of the best model; R^2^ = the explained variance of each model.

**Table 6:** The best models from the Bayesian linear regression for somatic symptoms in the non-clinical sample.

	p(M)	p(M|*D*)	BFM*j*	BF_01_	R^2^
Worry + Social Skills	0.00	0.51	176.43	2.60 × 10^−15^	0.09
Worry	0.00	0.07	4.16	5.69 × 10^−14^	0.08
Gender + Social Skills + Worry	0.00	0.06	19.28	1.24 × 10^−14^	0.09
Expression and Evaluation of Emotion to Self + Social Skills + Worry	0.00	0.05	17.05	1.39 × 10^−14^	0.09
Age + Social Skills + Worry	0.00	0.04	13.55	1.73 × 10^−14^	0.09
Evaluation and Expression of Emotion to Others + Social Skills + Worry	0.00	0.04	11.48	2.03 × 10^−14^	0.09
Social Skills + Optimism/Mood Regulation + Worry	0.00	0.03	10.56	2.20 × 10^−14^	0.09
Expression and Evaluation of Emotion to Self + Worry	0.00	0.01	2.65	8.53 × 10^−14^	0.08
Age + Worry + Expression and Evaluation of Emotion to Self + Social Skills + Worry	0.00	0.01	3.50	6.47 × 10^−14^	0.09

Note: p(M) = prior model probabilities; p(M|*D*) = posterior model probabilities; BFM*j* = the change from prior to posterior model odds.

**Table 7:** The best models from the Bayesian linear regression for affective symptoms in the subclinical sample with elevated depressive symptoms.

	p(M)	p(M|*D*)	BFM*j*	BF_01_	R^2^
Worry	0.01	0.05	3.32	1.04	0.03
Expression and Evaluation of Emotion to Self	0.01	0.03	1.93	1.75	0.02
Gender + Age + Expression and Evaluation of Emotion to Self + Expression and Evaluation of Emotion to Others + Social Skills + Optimism/Mood Regulation + Worry	0.12	0.02	0.20	14.78	0.09
Gender	0.01	0.02	1.59	2.12	0.01
Expression and Evaluation of Emotion to Others	0.01	0.02	1.46	2.30	0.01
Worry + Expression and Evaluation of Emotion to Self + Expression and Evaluation of Emotion to Others	0.00	0.01	3.04	1.11	0.05
Age	0.01	0.01	0.96	3.45	0.00
Optimism/Mood Regulation	0.01	0.01	0.70	4.71	0.00
Social Skills	0.01	0.01	0.67	4.93	0.00

Note: p(M) = prior model probabilities; p(M|*D*) = posterior model probabilities; BFM*j* = the change from prior to posterior model odds.

**Table 8:** The best models from the Bayesian linear regression for somatic symptoms in the subclinical sample with elevated depressive symptoms.

	p(M)	p(M|*D*)	BFM*j*	BF_01_	R^2^
Gender + Age + Expression and Evaluation of Emotion to Self + Expression and Evaluation of Emotion to Others + Social Skills + Optimism/Mood Regulation + Worry	0.12	0.09	0.73	0.95	0.15
Age + Worry + Expression and Evaluation of Emotion to Self + Worry	0.00	0.04	11.77	0.06	0.12
Expression and Evaluation of Emotion to Self	0.01	0.03	2.14	0.34	0.05
Expression and Evaluation of Emotion to Self + Age+ Expression and Evaluation of Emotion to Others + Social Skills + Optimism/Mood Regulation + Worry	0.0	0.03	1.79	0.41	0.15
Age + Expression and Evaluation of Emotion to Self	0.00	0.02	4.22	0.17	0.08
Age + Expression and Evaluation of Emotion to Self + Expression and Evaluation of Emotion to Others + Social Skills + Worry	0.00	0.02	6.75	0.11	0.13
Gender + Age + Expression and Evaluation of Emotion to Self + Expression and Evaluation of Emotion to Others + Social Skills + Worry	0.01	0.02	1.20	0.60	0.14
Gender + Age + Expression and Evaluation of Emotion to Self + Social Skills + Optimism/Mood Regulation + Worry	0.01	0.02	1.19	0.61	0.14
Optimism/Mood Regulation + Worry	0.00	0.02	3.49	0.21	0.08

Note: p(M) = prior model probabilities; p(M|*D*) = posterior model probabilities; BFM*j* = the change from prior to posterior model odds.

**Table 9:** Bayesian Independent Samples *T*-Test.

	BF_01_
1. Evaluation and Expression of Emotion to Self	1.08 × 10^−4^
2. Evaluation and Expression of Emotion to Others	6.19
3. Social Skills	2.72 × 10^−9^
4. Optimism/Mood Regulation	6.08 × 10^−4^
5. Affective symptoms	1.47 × 10^−148^
6. Somatic symptoms	1.26 × 10^−11^
7. Worry	2.21 × 10^−24^

## Data Availability

Data are available through the corresponding author upon reasonable request.

## Supplementary Materials

The following supporting information can be downloaded at: https://www.mdpi.com/article/10.3390/ejihpe16040048/s1.
