# Supplementary material for: The Role of Worry and Emotional Intelligence in Depression in a Non-Clinical and Subclinical Sample
Source: Eur J Investig Health Psychol Educ. 2026 Mar 28;16(4):48. doi: 10.3390/ejihpe16040048 (PMC13114789; doi:10.3390/ejihpe16040048)
Supplement: Supplementary file 1 [file ejihpe-16-00048-s001.zip › ejihpe-3948083-supplementary.pdf]

Table S1. *Model averaged posterior summary for linear regression coefficients of the Affective symptoms in the non-clinical sample.*

Coefficient	P(incl)	P(excl)	P(incl|data)	$BF_{inclusion}$	Mean	SD	95% Credible Interval	
							Lower	Upper
Gender	0.500	0.500	0.555	1.249	-0.254	0.277	-0.715	0.000
Age	0.500	0.500	0.282	0.392	-0.003	0.007	-0.021	1.945×10^{-4}
Worry	0.500	0.500	1.000	$6.344 \times 10^{+17}$	0.092	0.010	0.073	0.109
Expression and Evaluation of Emotion to Self	0.500	0.500	1.000	10541.049	-0.109	0.022	-0.151	-0.071
Evaluation and Expression of Emotion to Others	0.500	0.500	0.310	0.450	0.013	0.024	0.000	0.070
Social Skills	0.500	0.500	0.196	0.244	-0.006	0.020	-0.075	0.002
Optimism/Mood Regulation	0.500	0.500	0.156	0.185	-8.103×10^{-4}	0.018	-0.035	0.040

Note. p(incl) = the prior inclusion probability; p(incl|data) = the posterior inclusion probability; BFinclusion = the change from prior to posterior inclusion odds; M = posterior Mean of the parameter after model averaging; SD = posterior Standard deviation of the parameter after model averaging.

Table S2. *Model averaged posterior summary for linear regression coefficients of the Somatic symptoms in the non-clinical sample.*

Coefficient	P(incl)	P(excl)	P(incl|data)	$BF_{inclusion}$	Mean	SD	95% Credible Interval	
							Lower	Upper
Gender	0.500	0.500	0.144	0.168	0.035	0.124	-0.057	0.357
Age	0.500	0.500	0.114	0.128	6.950×10^{-4}	0.004	-0.003	0.009
Expression and Evaluation of Emotion to Self	0.500	0.500	0.150	0.177	-0.004	0.014	-0.055	0.000
Evaluation and Expression of Emotion to Others	0.500	0.500	0.106	0.119	-0.001	0.011	-0.041	0.003
Social Skills	0.500	0.500	0.887	7.834	-0.108	0.053	-0.179	0.000
Optimism/Mood Regulation	0.500	0.500	0.100	0.112	5.028×10^{-4}	0.015	-0.027	0.016
Worry	0.500	0.500	1.000	$2.837\times10^{+11}$	0.081	0.010	0.060	0.102

Note. p(incl) = the prior inclusion probability; p(incl | data) = the posterior inclusion probability; BFinclusion = the change from prior to posterior inclusion odds; M = posterior Mean of the parameter after model averaging; SD = posterior Standard deviation of the parameter after model averaging.

Table S3. *Model averaged posterior summary for linear regression coefficients of the Affective symptoms in the subclinical sample with elevated depressive symptoms.*

Coefficient	P(incl)	P(excl)	P(incl|data)	$BF_{inclusion}$	Mean	SD	95% Credible Interval Lower	95% Credible Interval Upper
Gender	0.500	0.500	0.232	0.302	-0.243	0.630	-2.080	0.080
Age	0.500	0.500	0.221	0.284	0.009	0.024	-0.001	0.082
Expression and Evaluation of Emotion to Self	0.500	0.500	0.300	0.428	-0.036	0.068	-0.207	0.001
Expression and Evaluation of Emotion to Others	0.500	0.500	0.264	0.360	0.032	0.069	-0.005	0.219
Social Skills	0.500	0.500	0.165	0.198	8.057×10^{-4}	0.053	-0.162	0.132
Optimism/Mood Regulation	0.500	0.500	0.175	0.211	-0.009	0.061	-0.171	0.107
Worry	0.500	0.500	0.294	0.416	0.015	0.030	-3.179×10^{-4}	0.101

Note. p(incl) = the prior inclusion probability; p(incl|data) = the posterior inclusion probability; BFinclusion = the change from prior to posterior inclusion odds; M = posterior Mean of the parameter after model averaging; SD = posterior Standard deviation of the parameter after model averaging.

Table S4. *Model averaged posterior summary for linear regression coefficients of the Somatic symptoms in the subclinical sample with elevated depressive symptoms.*

							95% Credible Interval	
Coefficient	P(incl)	P(excl)	P(incl|data)	$BF_{inclusion}$	Mean	SD	Lower	Upper
Gender	0.500	0.500	0.336	0.506	0.067	0.447	-0.711	1.399
Age	0.500	0.500	0.658	1.928	0.041	0.038	-6.130×10^{-4}	0.113
Expression and Evaluation of Emotion to Self	0.500	0.500	0.596	1.477	-0.060	0.070	-0.221	0.010
Expression and Evaluation of Emotion to Others	0.500	0.500	0.367	0.580	0.018	0.051	-0.046	0.174
Social Skills	0.500	0.500	0.458	0.846	-0.057	0.095	-0.269	0.064
Optimism/Mood Regulation	0.500	0.500	0.475	0.904	-0.061	0.100	-0.312	0.065
Worry	0.500	0.500	0.661	1.950	0.037	0.035	-0.002	0.101

Note. p(incl) = the prior inclusion probability; p(incl | data) = the posterior inclusion probability; BFinclusion = the change from prior to posterior inclusion odds; M = posterior Mean of the parameter after model averaging; SD = posterior Standard deviation of the parameter after model averaging.
